# Computational Analysis of Clinical and Molecular Markers and New Theranostic Possibilities in Primary Open-Angle Glaucoma

**DOI:** 10.3390/jcm9093032

**Published:** 2020-09-21

**Authors:** María D. Pinazo-Durán, José J. García-Medina, José M. Bolarín, Silvia M. Sanz-González, Mar Valero-Vello, Javier Abellán-Abenza, Vicente Zanón-Moreno, Javier Moreno-Montañés

**Affiliations:** 1 Ophthalmic Research Unit “Santiago Grisolía”/FISABIO and Cellular and Molecular Ophthalmo-Biology Group of the University of Valencia, 46010 Valencia, Spain; jj.garciamedina@um.es (J.J.G.-M.); sanz-gonzalez.sm@gmail.com (S.M.S.-G.); vavema@alumni.uv.es (M.V.-V.); vczanon@universidadviu.com (V.Z.-M.); 2 Researchers of the Spanish Net of Ophthalmic Research “OFTARED” RD16/0008/0022, of the Institute of Health Carlos III, 28029 Madrid, Spain; jmoreno@unav.es; 3 Department of Ophthalmology at the University Hospital “Morales Meseguer” and Department of Ophthalmology at the Faculty of Medicine, University of Murcia, 30008 Murcia, Spain; 4 Center of Information and Communication Techniques (CENTIC), 30100 Murcia, Spain; josemiguel.bolarin@centic.es (J.M.B.); javier.abellan@centic.es (J.A.-A.); 5 Area of Health, Valencian International University, 46002 Valencia, Spain; 6 Department of Ophthalmology at the Clínica Universidad de Navarra, 31008 Pamplona, Spain

**Keywords:** primary open-angle glaucoma, pathogenesis, oxidative stress, inflammation, apoptosis, neurodegeneration, theranostics

## Abstract

Primary open-angle glaucoma (POAG) is a paramount cause of irreversible visual disability worldwide. We focus on identifying clinical and molecular facts that may help elucidating the pathogenic mechanisms of the disease. By using ophthalmological approaches (biomicroscopy, ocular fundus, optical coherence tomography, and perimetry) and experimental tests (enzyme-linked immunosorbent assay (ELISA), high performance liquid chromatography (HPLC), and Western blot/immunoblotting) directed to evaluate the oxidative stress, inflammation, apoptosis, and neurodegeneration processes, we gather information to build a network of data to perform a computational bioinformatics analysis. Our results showed strong interaction of the above players and its downstream effectors in POAG pathogenesis. In conclusion, specific risk factors were identified, and molecules involved in multiple pathways were found in relation to anterior and posterior eye segment glaucoma changes, pointing to new theranostic challenges for better managing POAG progression.

## 1. Introduction

Primary open-angle glaucoma (POAG) is the most prevalent glaucoma type and the uppermost cause of irreversible blindness worldwide [1,2]. Current medical, laser, and surgical treatments are directed to lower intraocular pressure (IOP), but these are unable to cure the glaucomatous optic nerve degeneration. New strategies for POAG management include: (1) innovation in multimodal imaging (detection of apoptosing retinal cells (DARC), fluorescence lifetime imaging ophthalmoscope (FLIO), IOP telemetry, hydra optical coherence tomography (OCT), hyperspectral imaging, OCT elastography, OCT angiography (OCTA), oximetry, etc.); (2) electrophysiological testing of the retina and optic pathway (flash electroretinogram (ERG), electro-oculography (EOG), multifocal ERG (mfERG), multifocal visual evoked potential (mfVEP), oculo pattern ERG (PERG), visual evoked potential (VEP), etc.); and (3) emerging medical and surgical treatments (calcium channel blockers, a wide variety of potential neuroprotective natural products/drugs, small guanosine triphosphatase (GTPase) RHO-associated protein kinase inhibitors, new nano-based drug delivery approaches, contact lenses drug reservoir prototypes, and the challenging minimally invasive glaucoma surgery (MIGS) devices) [3,4,5,6].

Glaucoma biomarkers are directed to predict risk factors, to classify the stage of disease, to detail pathogenic mechanisms, to evaluate the progression as well as to monitor the therapeutics [3,6,7,8]. However, a variety of questions are currently difficult to ascertain when managing the disease. Clinical POAG hallmarks include anterior and posterior eye segment changes. Being the elevated IOP the main risk factor for developing/progressing the disease, medical therapy is the main line of POAG treatment. The major problem of hypotensive medical therapy is the poor adherence of the POAG patients. Existing neuroprotective therapies are still considerably limited. Comprehensive neuroprotection/neuroregeneration and other promising discoveries are the ultimate frontier in glaucoma treatment. In addition, an important inconvenience of glaucoma surgery is the uncontrolled wound healing process and related complications [3,4,5,6,7,8].

Biochemical and molecular biomarkers (BMB) for POAG have been widely reported, such as a variety of indicators of immune response, oxidative stress (OS) and mitochondrial failure, extracellular matrix alterations, vascular damage, and cell death [3,9,10,11,12,13,14]. Most of these markers strongly indicate adaptive/protective/repairing failure responses from the cells and tissues, as well as those reflecting the damage arising from OS [3,9,10,11,12]. In order to better diagnose and treat POAG, the differential signature of biofluids has been largely utilized in humans and experimental glaucoma models [13,15,16,17,18]. Information about these markers has generated large amounts of data. However, managing these large data sets requires accurate assessments by forecasting bioinformatics tools [19].

Theranostics is an innovative field in medical sciences, recently underscoring much attention [20]. It takes advantage of a combination of particular targeted diagnostic probes and specific targeted therapy, by using pivotal biological pathways, to achieve early diagnosis and treatment of human diseases [21,22,23,24]. In fact, theranostics is a newly proposed process of personalized diagnostic therapy for patients, with great unquestionably repercussion in social healthcare cost.

Presumably, supra-molecular targeted theranostics may have a successful role to better manage outstanding pathologies. Therefore, we deal with defining clinical and molecular-genetic facts that enlighten the POAG risk factors and pathogenic mechanisms to better diagnosing and treating glaucomatous eyes. By using ophthalmological imaging approaches (biomicroscopy (BMC), ocular fundus (OF), optical coherence tomography (OCT), and visual field (VF)) and experimental probes (enzyme-linked immunosorbent assay (ELISA), high performance liquid chromatography (HPLC), Western blot/immunoblotting), we collected large amounts of information. We build a data network to perform a computational analysis that may allow, for the first time, to reach new theranostic challenges for POAG.

## 2. Materials and Methods

### 2.1. Study Design

This research study received the Institutional Board approval of the main study center (Valencia, ref: 132/2018), as well as by the Ethics and Clinical Research Committees of the participating hospitals and research centers. All procedures adhered to the principles outlined in the Declaration of Helsinki for research involving human subjects, the European Commission rules for research, and the Association for Research in Vision and Ophthalmology (ARVO) guidelines regarding the ethical use of human subjects in research. All experiments were performed under the informed consent of the participants signed at any time.

We devised a large experimentation in order to compare a compendium of our own archive of datasets from POAG patients and healthy individuals, meaning over different biological systems in order to gather information on the major risk factors, pathological mechanisms, and current therapy for improving knowledge on POAG early diagnosis and treatment. In subsequent sections, we depict the participant characteristics, ophthalmological examination protocols, sampling procedures, and the experimental approaches used along with the data analysis, correlation measures, and performance metrics that we have carried out in this study.

### 2.2. Proceedings

The present work was a cohort study for analyzing the descriptive statistics and exploring the risk factors, biochemical and molecular facts, and therapeutic characteristics that might help in predicting glaucoma worsening in a Spanish population with POAG, using healthy individuals as a control group (CG).

#### 2.2.1. Study Population

A total of 632 Caucasian individuals of both sexes aged ≥ 40 years were considered for this study and distributed into 442 POAG patients and 190 CG subjects. Cases and controls have been classified according to the inclusion and exclusion criteria, as listed in Table 1.

POAG were identified by gonioscopy presenting 3–4 grades of Shaffer, an IOP ≥ 21 mmHg (at least 3 different days), and signs of glaucomatous optic nerve damage (cupping, notching, and/or hemorrhages in the optic nerve head) with typical glaucomatous defects in the standard automated perimetry (stage 1–4 in the Glaucoma Staging System [25] with mean deviation ≤−12 decibels according to the Hoddap, Parrish, and Anderson classification [26]. Data were collected from both eyes (884 eyes) regardless of the affectation, and finally, 736 glaucomatous eyes were considered for this study.

Control subjects presented IOP ≤ 21 mmHg, a normal standard automated perimetry, with no signs of glaucomatous damage in the optic nerve. Both eyes of each participant were included in this analysis (380 eyes).

Each ophthalmologist reported the corresponding participant data (anonymously) by means of a self-design report form that was entered and processed by the Microsoft Excel spreadsheet program for Windows.

#### 2.2.2. Demographics and Participant Characteristics

Demographic data and participant characteristics were registered from each individual: age, gender, as well as the family history of POAG. Nutrition facts were also investigated and the level of adherence to the Mediterranean diet (MedDiet) standards (nutritional and lifestyle pattern based on the traditional dietary habits of Greece, Italy, and Spain) were registered. The latter were addressed by means of the brief 14-item validated questionnaire [27]. The adherence to the MedDiet was defined through scores determining the conformity of the dietary pattern of the studied population with the traditional Mediterranean dietary pattern. Values of 0–1 were assigned to each dietary component by using as cut offs the overall sex specific medians among the study participants. Specifically, people whose consumption of components considered to be part of a Mediterranean diet (vegetables, fruits, legumes, cereals, fish, and a moderate intake of red wine during meals) was above the median consumption of the population were assigned a value of one, whereas a value of zero was given to those with consumptions below the median. By contrast, people whose consumption of components presumed not to form part of a Mediterranean diet (red and processed meats, dairy products) was above the median consumption of the population had a value of zero assigned, and the others had a value of one.

#### 2.2.3. Clinical Variables

POAG duration was considered as the lead time between the diagnosis of the disease and the first review of the medical history of the glaucoma patient, and the data were recorded.

Types of used hypotensive eye drops were categorized and recorded, according to the following distinction: (a) prostaglandin analogues (PA); (b) beta-blockers (BB); (c) fixed combination of PA + BB; (d) alpha-agonists (AA); (e) carbonic anhydrase inhibitors (CAI); (f) fixed combination of BB + CAI; (g) fixed combination of BB + AA.

Previous history of glaucoma surgery and the type of surgical procedure were also recorded from each glaucomatous patient, as follows: (a) trabeculectomy, (b) non-perforating deep sclerectomy (NPDS), (c) MIGS, (d) combined phacoemulsification and glaucoma surgery.

Ophthalmological examination included: best corrected visual acuity (BCVA) in decimal scale, IOP measurement, slit-lamp biomicroscopy of the anterior eye segment and media, gonioscopy, dilated stereoscopic fundus examination, VF performance, using the 24–2 Swedish interactive threshold algorithm (Humphrey field analyzer, Carl Zeiss Meditec, Inc., Madrid, Spain), and optic disc analysis using the Cirrus Spectral domain OCT 5000 (Carl Zeiss Meditec, Inc., Madrid, Spain) with special interest in detecting the retinal nerve fiber layer (RNFL) changes. The reference of the software of this tool was 11.0.0.29946.

#### 2.2.4. Sample Handling

Biological samples were collected according to their respective processing protocols. Blood, aqueous humor, or tears were alternatively collected from the participants.

Blood was collected from venipuncture of the antecubital vein under fasting conditions (10−11 h). Vacutainer^®^ (Becton, Dickinson and Co., Franklin Lakes, NJ, USA) blood collection tubes were used and immediately labeled. Plasma was obtained by centrifugation of whole blood samples, aliquoted, labeled, and stored at −80 °C until processing, as reported [28].

Aqueous humor samples were collected at the beginning of surgical maneuvers. The type of surgery was directly related to the study group, as follows: for the POAG group, trabeculectomy or combined glaucoma surgery/phacoemulsification proceedings; and for the comparative group, phacoemulsification. An anterior chamber 27 G Rycroft cannula angled 45 degrees was introduced in the anterior chamber through the corneal incision, under the operating microscope. Approximately 150  μL were obtained from each eye through the 1 mL microsyringe attached to the cannula (for avoiding anterior chamber collapse), immediately deposited in cryotubes, labeled and frozen at −80 °C until processing, as previously published [10,29].

Reflex tears were collected from the inferior tear meniscus of each eye, by gently rubbing in the inferior meniscus of both eyelids with a microglass pipette, as previously described [30]. Reflex tears (20–30 μL) were deposited in a mini Eppendorf, labeled and stored at −80 °C until processing.

#### 2.2.5. Biochemical and Molecular Variables

##### Oxidative/Nitrosative Stress

Malondialdehyde (MDA) was determined by the MDA-thiobarbituric acid reactive substances (TBARS) assay [29,31,32]. Briefly, aqueous humor and plasma samples from POAG patients, cataract patients, or healthy controls were treated with HCl and SDS. The MDA present in samples reacts with TBA and the fluorescent complex formed is measured in a Fluoroskan^®^ Ascent FL (Thermo Electron Corporation, Philadelphia, PA, USA).

MDA was also determined by HPLC in blood and aqueous humor of POAG and cataract patients, using a Vydac 250 × 4.6 mm, 5 μm particle-size column with its own guard column (Eka Chemicals AB, Bohus, Sweden), and a surveyor PDA detector (ThermoFinnigan Italia) with a wavelength range of 200–300 nm [33,34].

Superoxide dismutase was determined in aqueous humor of POAG patients and cataract patients using the superoxide dismutase (Ransod) assay (Randox Labs, Barcelona, Spain). We followed the manufacturer’s instructions for performing the assay, as in our previous work [35].

Glutathione peroxidase (GPx) was measured in plasma from POAG patients and healthy controls using the glutathione peroxidase assay kit (Cayman Chemical Co.), following the manufacturer’s instructions and our previous assays [36].

The total antioxidant capacity (TAC) was analyzed in aqueous humor from POAG and cataract patients by means of the total antioxidant status kit (Randox Labs), following the manufacturer’s instructions [29,35].

We also used the antioxidant assay kit (Cayman Chemical Co., Ann Arbor, MI, USA) to analyze TAS in plasma samples from POAG patients and healthy controls [29,32,35,36].

In addition, we used the oxygen-radical absorbance capacity method (ORAC) to determine the TAS in blood and aqueous humor from POAG and cataract patients [34]. This method measures the antioxidant capacity of a substance according to its ability to inhibit or delay β-phycoerythrin peroxidation.

The concentration of the antioxidant vit C was analyzed in plasma samples from POAG patients and healthy controls by high performance liquid chromatography (HPLC) following the method described by Li [37] using a Shimadzu HPLC system (Shimadzu Scientific Instruments, Columbia, MD, USA) equipped with a 5 µM YMCPack ODS-AQ column (Waters Corp., Milford, MA, USA) and a Coulochem III electrochemical detector (ESA, Chelmsford, MA, USA), under reversed phase conditions. The volume of sample injection was 5 µL and compounds were eluted over an 18-min runtime at a flow rate of 0.6 mL/min [36,38].

Nitric oxide (NO) concentration in aqueous humor from POAG and cataract patients was determined by means of the total NO/nitrite/nitrate assay (R&D Systems, Inc., Minneapolis, MN, USA). Most of the NO in the body is in the form of nitrite and nitrate. Therefore, this method indirectly quantifies nitric oxide based on the enzymatic conversion of nitrate to nitrite by nitrate reductase, followed by a by colorimetric detection of nitrite as an azo dye product of the Griess reaction [39].

##### Inflammation and Immune Response

Cytokines and chemokines were determined in tears of POAG patients and healthy controls by the Luminex^®^ R-200 multiplex system [30,40]. We analyzed the following molecules: interleukin (IL)-1β, IL-2, IL-4, IL-5, IL-6, IL-7, IL-8, IL-10, and IL-12; tumor necrosis factor alpha (TNFα); vascular endothelial growth factor (VEGF); granulocyte-macrophage colony-stimulating factor (GM-CSF); and interferon gamma (IFγ). Concentrations were calculated automatically by the BioPlex Manager software using a standard curve derived from a recombinant cytokine standard.

Interleukin 6 also was measured in aqueous humor and plasma samples from POAG and cataract patients using the interleukin-6 (human) ELISA kit (Cayman Chemical Co. Ann Arbor, MI, USA), following the manufacturer’s instructions [41]. This method is based on a double-antibody “sandwich” technique using a monoclonal antibody specific for IL-6 and an acetylcholinesterase: Fab’ conjugate, and measuring the enzymatic activity of acetylcholinesterase at 412 nm. The concentration of the IL-6 is calculated from the bound conjugate, since it is proportional to the acetylcholinesterase concentration.

##### Apoptosis Assays

Caspase-3 (CAS3) and poly (ADP-ribose) polymerase 1 (PARP1) levels were determined in aqueous humor and plasma samples from POAG and cataract patients by Western blot and immunoblotting procedures, as described by Chandra and Tang [42].

First, we analyzed the concentration of total proteins following a procedure similar to Lowry’s [43]. Then, 30 µg protein were loaded on a 4–12% Bis-Tris gel and proteins are separated by electrophoresis. After, we carried out the transfer to the nitrocellulose membrane and finally CAS3 and PARP1 were detected by immunoblotting using specific antibodies (PARP H-250, Santa Cruz Biotechnology Inc., Santa Cruz, CA, USA; Cleaved Caspase-3, Cell Signaling Technology, Danvers, MA, USA). The band optical density was determined and analyzed by Scion Image Analysis (Scion Corp., Frederick, MD, USA).

##### Neuroprotection Status

Brain-derived neurotrophic factor (BDNF) was determined in aqueous humor samples from patients with POAG and patients with cataracts, using the human BDNF immunoassay Quantikine kit (R&D Systems, Inc.), following the manufacturer’s instructions and previous reports [44].

##### Neurotransmitter Determinations

We analyzed the concentration of serotonin (5-hydroxytriptamine, 5 HT) as well as dopamine in aqueous humor of POAG and cataract patients by HPLC with electrochemical detection, according to a modified method of Ali et al. [45]. The assay was carried out using a Gilson Medical Electronics HPLC system (Middleton, WI, USA) with a Supelcosil LC 7.5 × 4.6 cm, 3 mm column (Supelco; Sigma-Aldrich, Bellefonte, PA, USA), and a LC142 electrochemical detector under reversed phase conditions. Compounds were eluted isocratically over an 18 min runtime at a flow rate of 1 mL/min. Sample injection was 20 mL, and the electrochemical detector was recorded with a glassy carbon working electrode set at + 0.75 V.

#### 2.2.6. Bioinformatics

Large-scale real clinical data were simultaneously collected from the archives of the study centers in order to construct the dataset. Data from 442 POAG patients (736 eyes) and 190 CG individuals (380 eyes) were collected in sheaths of the Microsoft Excel program. One important point to consider is that statistical processing has been done for the two eyes of each participant, just considering that only eyes with initial glaucoma stage, according to the Glaucoma Staging System [25] and the classical classification by Hoddap, Parrish, and Anderson [26], were suitable for the purpose of the study, and that the correlation between the RE and the LE data, in this sense, was expected to be high. All the described experiments were performed in duplicate for each sample. Quantitative variables where summarized using mean ± standard deviation (SD). Categorical variables were expressed as percentages. The Shapiro–Wilk test was used to assess for normality in each variable. The Student *t* test was used to compare normally distributed variables among controls and glaucoma patients, whilst for non-normally distributed variables, the Mann–Whitney test was used. Differences in qualitative variables between both groups where compared using Fisher’s test. The level of statistical significance was set at *p* < 0.05. All statistical analyses were performed using R Statistics v4.0.0 (R Foundation for Statistical Computing, Vienna, Austria).

## 3. Results

Accurate information for the main purpose of this study, to raise POAG awareness, was obtained from our recorded data sets from 632 participants that constituted our study cohort. Data collection was organized into those pertaining to 442 POAG patients and 190 CG individuals, in order to integrate multiple datasets by data-merging methods.

We devised a large experimentation in order to compare a compendium of demographics, participant characteristics, risk factors, ophthalmologic and molecular datasets from POAG patients and healthy controls, meaning over different biological systems (plasma, aqueous humor, tears) in order to gather information on the major risk factors, pathological mechanisms, and current therapy for improving knowledge on POAG early diagnosis and treatment. In subsequent sections, we depict extensively the above collected data that were analyzed by bioinformatics, and the results are exposed below.

### 3.1. Demographics and Participant Characteristics

Our study population was composed of 442 POAG patients (45% men and 55% women) and 190 healthy controls (40% men and 60% women). Mean age of our cohort was 67 ± 13 years for the POAG patients and 52 (16) years for the CG individuals. Mean glaucoma duration was 9 ± 7 years. Familial POAG history was present in 49% of the affected patients. In our study course, we have detected that the mean of the 14-item MedDiet answers were noticeably lower in the POAG patients than in the CG (7.1 ± 3 and 8.5 ± 2), respectively. Furthermore, more healthy individuals than glaucomatous patients reached 10 points within the adherence to the MedDiet score.

### 3.2. Ophthalmologic Evaluation

**Figure 1:**
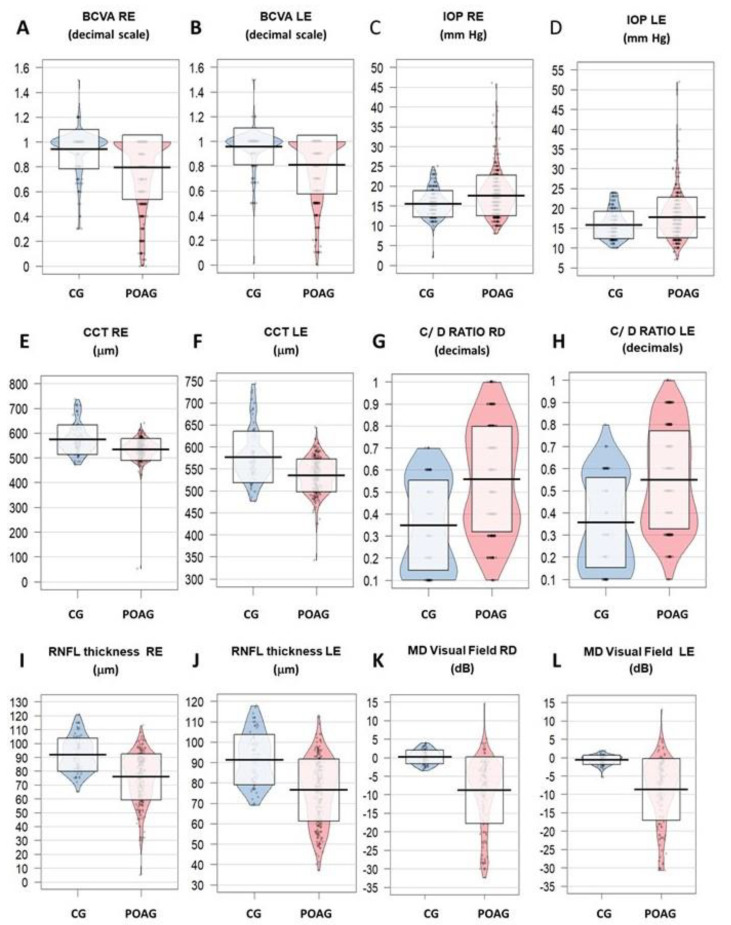
Ophthalmological evaluation of the study participants. (**A**,**B**) show the best corrected visual acuity (BCVA) (expressed in decimals; see the LogMAR correspondence in the text) in the right and left eyes. (**C**,**D**) indicate the IOP values (mmHg) in both eyes. In the (**E**,**F**), the CCT (in µm) from each eye was reflected. (**G**,**H**) display the C/D ratio in the eyes of the study participants. (**I**,**J**) show the RNFL thickness observed in the OCT examination from the participants eyes. Finally the (**K**,**L**) reflect the MD values obtained from the VF performance in both eyes. CG: control group; POAG: primary open-angle glaucoma; RE: right eye, LE: left eye; BCVA: best corrected visual acuity, IOP: intraocular pressure; CCT: central corneal thickness; C/D ratio: cup to disc ratio; RNFL: retinal nerve fiber layer; OCT: optic coherence tomography; MD: mean deviation; VF: visual field.

Data from the anterior and posterior eye segments examination were analyzed. The BCVA was significantly lower in both the RE (Figure 1A) and LE (Figure 1B) of the POAG patients as compared to the vision from the CG (*p* = 2.2 × 10^−16^ for each eye). The BCVA values expressed as the logarithm of the minimal angle of resolution (LogMAR) were: RE CG = 0.0; RE POAG = 0.0; LE CG = 0.0; LE POAG = 0.0). The IOP was significantly higher for each eye in the POAG group than in the CG (*p* = 2.6 × 10^−9^ and *p* = 9.2 × 10^−8^, respectively) (Figure 1C,D). Central corneal thickness (CCT) values were significantly lower for each eye in the POAG than in their counterparts (*p* = 5.5 × 10^−8^ and *p* = 5.8 × 10^−9^, respectively) (Figure 1E,F). Average cup/disc ratio was significantly higher for each eye in the POAG patients than in the CG (*p* = 2.1 × 10^−13^ and *p* = 2.4 × 10^−12^, respectively) (Figure 1G,H). The papillary OCT examination revealed that the average retinal nerve fiber layer (RNFL) thickness of each eye was significantly lower in the POAG patients respect to the CG (*p* = 2.2 × 10^−16^ for both eyes) (Figure 1I,J). The VF loss displays pathognomonic pattern in POAG (Bjerrum scotoma, paracentral scotoma, nasal step, or arcuate defect) corresponding to the damaged RNF. Ophthalmologists had to correlate findings from the visual field testing to better discriminate if the nerve fiber loss that appears on the OCT examination is the result of POAG or glaucoma masquerader. Data of our study population showed precise glaucoma changes in the ocular fundus, VF, and OCT examination of the POAG patients, respect the CG. Regarding the VF performance with each eye, measurement of the threshold values showed that the mean deviation (MD) was significantly lower in the POAG patients respect to the CG (*p* = 2.2 × 10^−16^ for both eyes), with negative values representing reduced sensitivity (Figure 1K,L).

Regarding the medical treatment, our data showed that the most frequent hypotensive eye drops utilized by our POAG sample patients were the PA (31%), followed by the BB (26%) and the fixed combination of PA + BB (21%). Therefore, in separate or as combination topical medication, the PA has been the most popular POAG treatment.

Independent of the glaucoma stage, the most frequent type of surgical procedure that our POAG patients underwent was the ab externo trabeculectomy (45%), followed by NPDS (32%) and the combined glaucoma and cataract surgery (22%).

### 3.3. Molecular Biomarkers

Data revealed a series of molecules presumptively involved in POAG pathogenesis. Characteristics and quantification of each molecule are enclosed below.

#### 3.3.1. Oxidative Stress

**Figure 2:**
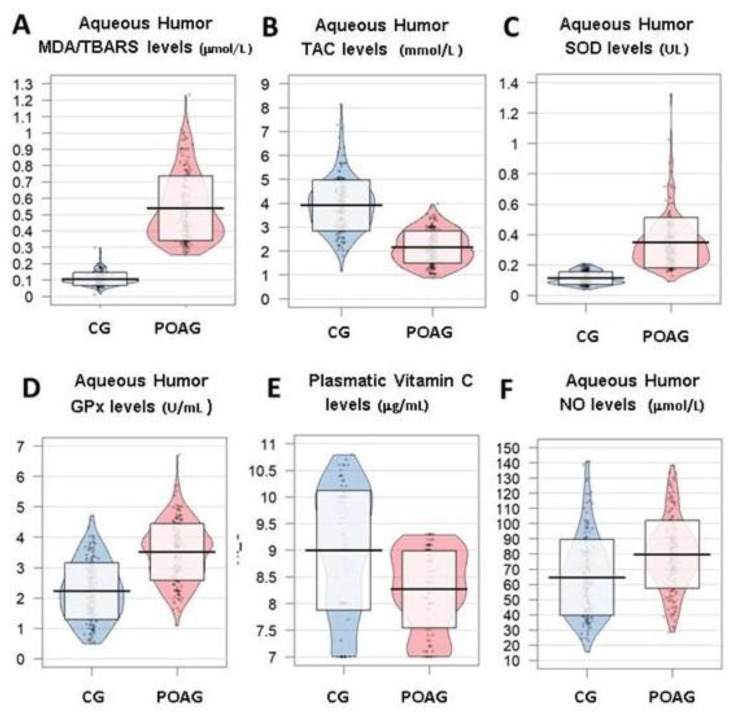
Oxidative stress markers in aqueous humor and plasma samples from the study participants. The OS overtime results in structural and functional eye damage. (**A**) Significantly higher MDA/TBARS expression levels (mol/L) in the aqueous humor from the POAG vs. the CG. (**B**) Significantly lower values of the TAC (nmol/L) in the aqueous humor from the POAG biosamples respect to those from the CG. (**C**) SOD expression shows significantly higher levels (UL) in the POAG aqueous humor as compared to the counterparts. (**D**) Significantly higher GPx levels were seen in the POAG aqueous humor vs. the CG. (**E**) Plasma vit C concentration (µg/mL) was significantly lower in the POAG vs. CG. (**F**) Aqueous humor NO levels (µmol/L) were significantly higher in the POAG patients respect to the healthy control participants. CG: control group (cataract subjects for aqueous humor comparative studies); POAG: primary open-angle glaucoma; MDA/TBARS: malondialdehyde/thiobarbituric acid reactive substances; TAC: total antioxidant capacity; SOD: superoxide dismutase; GPx: Glutathione peroxidase; NO: nitric oxide. *p* < 0.05 for all parameters in this figure.

When comparing with the samples from the comparative subjects (constituted by those undergoing surgery for non-complicated cataracts), MDA/TBARS (*p* = 2.2 × 10^−16^), SOD (*p* = 2.2 × 10^−16^), GPx (2.2 × 10^−16^) and NO (6.5 × 10^−10^) displayed significantly higher values in the aqueous humor of POAG patients (Figure 2A,C,D,F). In contrast, TAC was significantly lower in the glaucoma patients than in the controls (*p* = 2.2 × 10^−16^) (Figure 2B). In addition, the potent antioxidant vit C showed significantly lower plasma levels of the POAG group versus the control individuals (*p* = 1.2 × 10^−7^) (Figure 2E).

#### 3.3.2. Inflammation and Immune Response

**Figure 3:**
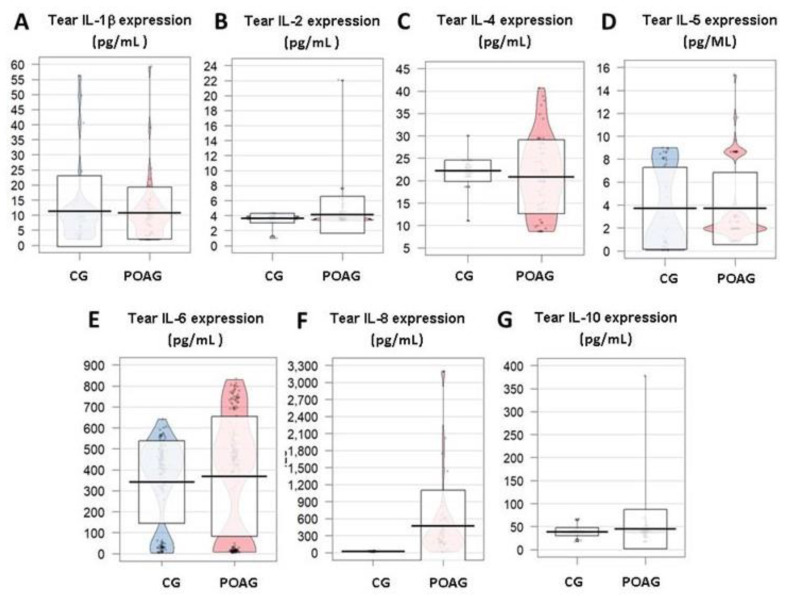
Cytokine expression in tears, aqueous humor, and plasma of glaucoma patients vs. the healthy controls. Differential expression profile of the IL-1β (**A**), IL-2 (**B**), IL-4 (**C**), IL-5 (**D**), IL-6 (**E**), IL-8 (**F**), and IL-10 (**G**) (as expressed in pg/mL). It is observed a statistically significant increase of the chemokine IL-8 as it can be seen in the (**G**), between the POAG vs. the CG tear samples. CG: control group; POAG: primary open-angle glaucoma; IL: interleukin.

In our study, noticeable changes in participants in the tear expression profile of the IL-1β, IL-2, IL-4, IL-5, IL-6, IL-8, and IL-10, with a statistically significant elevation of the chemokine IL-8 in the POAG patients respect to the CG (Figure 3A–G, respectively) were observed. Furthermore, an IL-6 significant elevation was detected in the aqueous humor of POAG patients respect to the CG.

**Figure 4:**
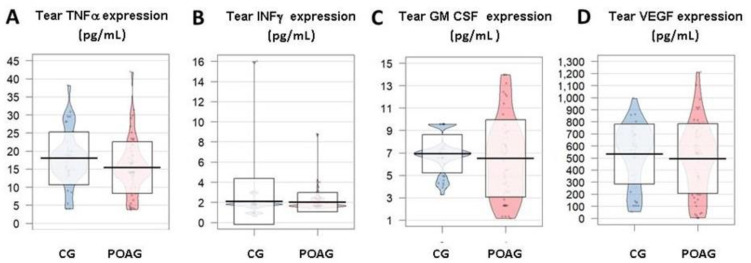
Set of inflammatory mediators TNFα (**A**), INF-g (**B**), GM CSF (**C**) and VEGF (**D**) as determined in biological samples of the study participants (expressed in pg/mL). Only the tear expression of TNFα, as reflected in the (**B**) was significantly different between the two study groups (*p* < 0.05). CG: control group; POAG: primary open-angle glaucoma; TNFα: tumor necrosis factorα; INFγ: interferonγ; GM-CSF: granulocyte-macrophage colony stimulating factor; VEGF: vascular endothelial growth factor.

Being an important mediator of both the acute and chronic inflammation, TNFα has shown an important decrease in the tear expression patterns of the POAG patients as compared to the CG (*p* = 0.080) (Figure 4A).

We have found a notable decrease of the INFγ in tears from POAG patients respect to tears from the CG (*p* = 0.872) (Figure 4B).

In our POAG patients, increased GM CSF tear expression, as compared to the CG (*p* = 0.410) was observed (Figure 4C).

Despite that different signaling driven by VEGF in the pathogenesis of POAG remains unclear, a decrease in VEGF tear expression in the POAG patients respect to the CG was detected (*p* = 0.475) (Figure 4D) in our study participants.

#### 3.3.3. Apoptosis

**Figure 5:**
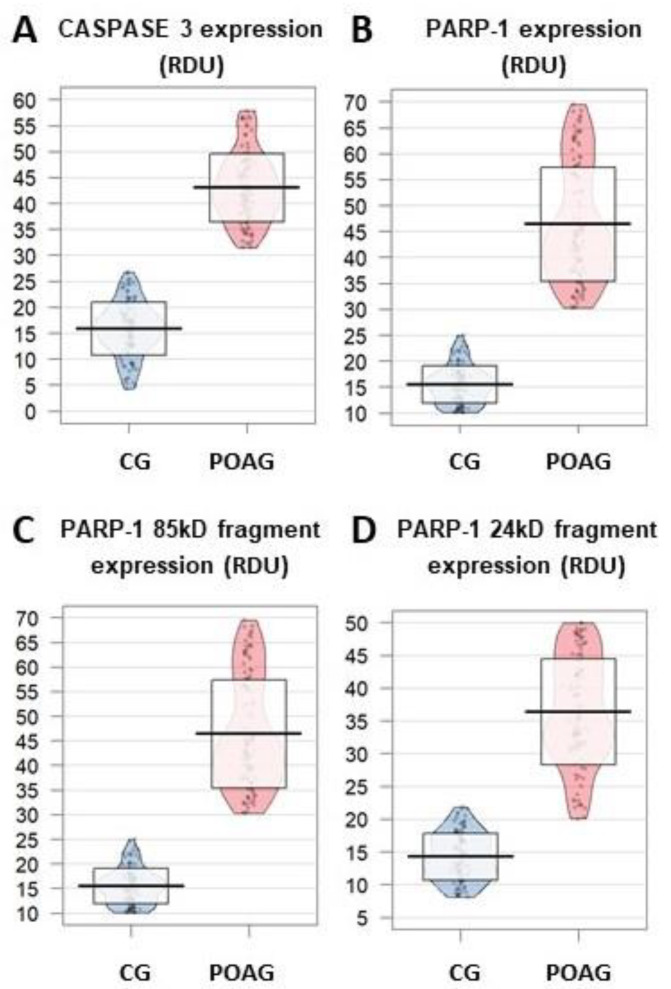
Differential expression profile (relative densitometric units) in the aqueous humor from the glaucoma vs. the control participants. The CASP3 (**A**) and PARP-1 (**B**) were significantly higher in the POAG vs. the CG. Comparison of the expression profile of the PARP-1 85 kD and 24 kD fragments between the aqueous humor from the POAG and the the CG are reflected in (**C**) and (**D**). CASP3: caspase 3; CG: control group (cataract subjects forming the comparative group); POAG: primary open-angle glaucoma; RDU: relative densitometric units (laser densitometry); PARP-1: poly (ADP-ribose) polymerase.

CASP 3 levels appeared to be significantly higher in the POAG group than in the CG (formed by the comparative cataract subjects) (*p* = 2.2 × 10^−16^) (Figure 5A).

Increased PARP1 expression was detected in the POAG patients respect to the CG ones (Figure 5B). Specifically, both the 85 kDa and 24 kDa fragments displayed significantly higher expression in the POAG group than in the CG (*p* = 2.2 × 10^−16^ for both fragments) (Figure 5C,D).

#### 3.3.4. Neurodegeneration/Neuroprotection

**Figure 6:**
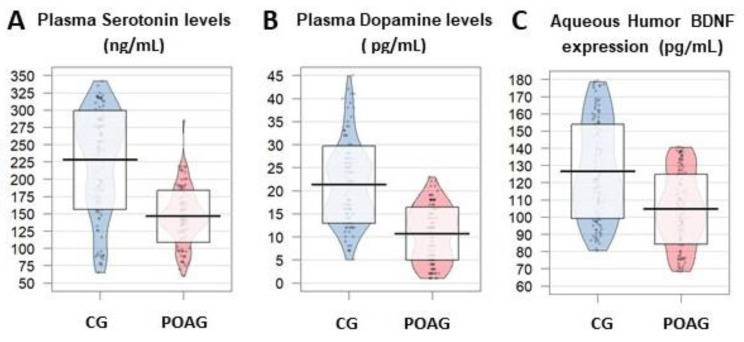
Neuroprotectants and neurotransmitters in the study participants. (**A**,**B**) Plasma serotonin and dopamine expression (ng/mL) was significantly lower in samples of the POAG patients than in the CG. (**C**) Brain Derived Neurotrophic Factor (BDNF) aqueous humor expression (pg/mL) was significantly lower in the POAG vs. the CG. CG: control group (cataract subjects as comparative group); POAG: primary open-angle glaucoma; BDNF: brain-derived neurotrophic factor. *p* < 0.05 for all parameters.

In the present cohort, significantly lower values of BDNF expression in the POAG patients than in the CG (*p* = 1.2 × 10^−12^) were found (Figure 6C).

#### 3.3.5. Neurotransmitters

Serotoninergic and dopaminergic catecholamines are essential neurotransmitter signaling players to a wide variety of CNS functions, including IOP regulation. In our cohort, serotonin and dopamine exhibited significantly lower plasma levels in the POAG patients than in the CG (*p* = 2.2 × 10^−16^ for both neurotransmitters) (Figure 6A,B)

**Figure 7:**
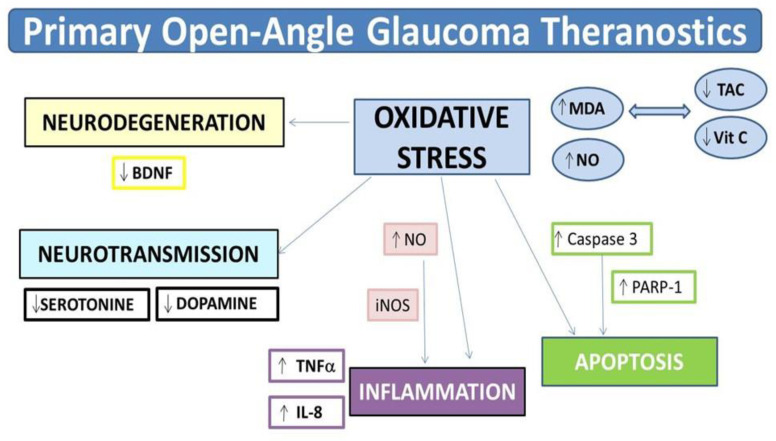
New diagnostic players and potential new therapies as primary-open-angle glaucoma theranostics. Excessive ROS generation/accumulation (superoxide anion (O2-), hydrogen peroxide (H_2_O_2_), and hydroxyl radical (OH)) selectively damage the lipids, proteins, and nucleic acids. MDA is an important lipid peroxidation byproduct. Elevated levels of pro-oxidants and decreased antioxidant activity spreads the oxidative injury. OS can induce activation of transcription factors, leading to gene expression involved in inflammation. In fact, OS stress activates the iNOS, a pivotal element of the transcription factor NF-kB pathway that controls the pro-inflammatory response by releasing among others the TNFα and IL-8, both significantly augmented in POAG. Serotonin and dopamine are really prone to autooxidation, and its toxic metabolites may contribute to increase neurodegeneration. Lower plasma levels of these neurotransmitters have been found in POAG. Glaucoma neurodegeneration is the result of the apoptotic death of the RGC. Lower levels of BDNF have also been identified in our POAG biosamples. ROS: reactive oxygen species; MDA: malondialdehyde; OS: oxidative stress; iNOS: inducible nitric oxide synthase; NF-κB: nuclear factor kappa B; TNFα: tumor necrosis factor alpha; IL-8: interleukin 8; RGC: retinal ganglion cells; BDNF: brain-derived neurotrophic factor.

Therefore, our data analyses allowed bioinformatics, researchers, and ophthalmologists to identify a set of potential POAG biomarkers. The term theranostics explains certain scientific developments that may lead to designing new therapeutic strategies for diverse pathologies. In the present work, we pinpoint specific aspects of the clinical and molecular POAG diagnosis that can be afforded by emerging analyses in fluids (Figure 7). Performing these assays, a variety of antioxidants, anti-inflammatory drugs, anti-apoptotic, and neuroprotectants may accurately be targeted to the underlying process (Figure 7) and the possibility remains that these can be controlled and followed by special tracers and imaging techniques.

Based on these approaches, we propose an open window to personalized potential biotherapeutic applications, to better manage POAG patients.

## 4. Discussion

To further analyze the link between the POAG risk factors and pathogenic mechanisms, we constructed a data network to perform a computational analysis. We built enough evidence that chronic IOP elevation is responsible of the RGC death and RNF loss, leading to optic nerve degeneration, originating in its course striking changes of a set of reactive molecules found in diverse body fluids (tears, aqueous humor, plasma), among them OS byproducts (MDA), inflammation (INF) mediators (TNFα, IL-6, IL-8), apoptotic effectors (such as the CASP3 and PARP-1), and finally the neurotrophic player BDNF. The characterization of the above molecules and their specific targeting by classic and/or emerging diagnostic and therapeutic approaches, open up innovating paradigms under the common name of theranostics with the main goal of improving eye and vision care in glaucoma patients.

Significant risk factors for POAG were similar in our study population (aging, glaucoma family history, elevated IOP, thinner CCT) than in other POAG-related population-based studies [7,46,47,48]. However, it is difficult to reach a powerful comparison of data from the epidemiologic studies, because of variations in the study design, tools and diagnostic criteria are potential confounding elements.

Normal aging involves a general reduction of RNFs. Peripapillary RNFL thickness was examined and quantified by OCT by Vianna et al. [49] and the data showed a clear age-related loss of neuroretinal parameters that may help explain the impairment detected in apparently controlled glaucoma-treated patients. These authors confirmed that glaucoma patients had faster rates of RNFL deterioration as compared to the controls [49]. In our glaucomatous participants, mean age was 67 ± 13 years, quite similar to other studies [7,46,47,48,49,50] and the RNFL thickness, quantified by OCT, was significantly lower than in its counterparts. The role of dietary components and dietary supplements in POAG has been widely investigated [51,52]. An observational study was conducted in the Spanish Canary Islands by Abreu et al. [53] to assess the adherence to the MedDiet in 100 patients affected by POAG. Overall, mean percentages of adhesion to the MedDiet of POAG patients were moderate (71% of the cases). In our study cohort, the cumulative analysis on the adherence to MedDiet scores were lower in the POAG patients than in the CG (7.1 ± 3 and 8.5 ± 2, respectively). These data mean that less POAG patient vs. the CG reached 10 points within the adherence to the MedDiet score. A meta-analysis of prospective cohort studies was performed by Sofi et al. [54] to evaluate the mortality in relation to adherence to the MedDiet. Interestingly, the authors found that a two point increase in the adherence score correlated with a decreased mortality risk. Overall, the study stated the beneficial role for health status of greater adherence to the MedDiet that was mainly reflected on significantly reduced cardiovascular or cancer mortality, as well as in the incidence of Alzheimer’s or Parkinson’s diseases. In spite of these, MedDiet did not reflect a homogeneous eating pattern, and subsequently, it has been taken into account that potential heterogeneity of scores within the 14-item questionnaire may exist. However, for most POAG patients, exhaustive recommendations on the modifiable risk factors may be done, although these lack entire warranty because of the contradictory data in the literature.

POAG persists as the main cause of irreversible blindness worldwide [1,2,8]. Hallmarks of the disease are the altered aqueous humor homeostasis that induces elevated IOP, which in turns lead to apoptotic RGC death and axonal dysfunction/RNF loss, reflected in the optic disc changes than can be observed in the ocular fundus examination (reaffirmed by OCT), and functional deficits (identified in the VF) [3,4,5,6,46,47,48,49,50,55].

Hypotensive eye drops are the treatment of choice of POAG. These drugs exert its function by decreasing the synthesis of the aqueous humor and/or increasing the aqueous humor elimination by classical or alternative pathways. The mechanism of lowering IOP for the PA is through facilitating the uveo-scleral aqueous humor outflow and for the BB by decreasing the aqueous humor production [3,6,7,8]. Both products are the most commonly used in POAG treatment. However, this is not effective at all, to slow down and regenerate the damaged retina and optic nerve. Laser or surgery is also not successful in this sense. Trabeculectomy is the most common surgery choice for reducing the IOP. However, MIGS is less effective, but safer than trabeculectomy [3,4,6]. Therefore, no cure exists for glaucoma neurodegeneration [56]. As in other clinical-based studies, in our POAG patients, the most frequent hypotensive eye drops were the PA (31%), followed by the BB (26%) and the fixed combination of PA + BB (21%), and the most frequent surgical procedure was the ab externo trabeculectomy (45%), followed by NPDS (32%) and the combined surgery of glaucoma and cataract (22%) [3,4,5,6,48,50,57].

Since the RGCs are so powerfully attacked in the glaucoma course, strategies may be directed to this issue.

Defined as the imbalance between pro-oxidants and antioxidants (with higher proportion of oxidative sources), the OS damages the cells and tissues. There is growing interest regarding key molecules that can be used as biomarkers of the onset or progression of OS and its downstream effectors. Mitochondria generate ATP by the electron transport chain and oxidative phosphorylation, thus producing (ROS). In normal conditions, mitochondria are well prepared to appropriately counteract ROS generation by the endogenous antioxidant defenses. Under pathological situations, the system fails, resulting in overdosing of pro-oxidants [3,9,10,12,30,31,32,33,34,35,36,58,59,60,61,62]. It has been reported that OS and mitochondrial failure are important processes in POAG pathogenesis [3,9,10,12,30,31,32,33,34,35,36,60,63]. The OS overtime induces structural and functional damage of the trabecular meshwork (TM), manifesting itself as the increased 8-OH-dG levels, as reported by Saccá et al. [64]. In this work, we have found significantly higher levels of MDA/TBARS in plasma and aqueous humor or POAG patients respect to the CG. In the contrary, we have detected significantly lower levels of TAC (in plasma and aqueous humor) and plasmatic vit C in the glaucomatous participants as compared to the CG, indicative of a marked oxidative stress situation leading pathology.

Another important player in POAG are the INF processes, as part of a biological response of the immune system towards the damaging agents, by means of both the innate and adaptative immunity [65]. Major players of the immune response are pro-inflammatory mediators, such as the following cytokines and chemokines: TNFα (mainly produced by macrophages), GM CSF (a pivotal regulator of granulocyte and macrophage populations, important constituents of the innate immunity), or type 1 IFNs (INFγ is an essential cytokine for innate/adaptive immunity, as important activator of macrophages and relevant inducer of the class II major histocompatibility complex molecule. Its aberrant expression is associated with a wide variety of inflammatory and autoimmune disorders). In fact, release of the above pro-inflammatory cytokines induces the activation of immune cells and the apparition of other cytokines. In this context, significant increase of TNFα and IL-6 has been detected in glaucomatous retinas, in response to oxidative overload [66]. VEGF is a promoter of AG in chronic inflammation, healing, and tumors. VEGF is also involved in the pathogenesis of a variety of diseases including blinding eye pathologies. In the present work, a decrease in VEGF tear expression in the POAG patients respect to the controls was found as expected. OS also plays a principal role in the activation of intracellular sensors, in order to defend the cells from damaging agents, the inflammasomes [67,68]. For addressing the role of INF in the pathogenesis of POAG, tears, aqueous humor, and blood samples were used to identify inflammatory biomarkers for glaucoma. Significant increase on the tear levels of the chemokine IL-8 in the POAG patients respect to the CG. Noticeable changes have also been detected in the differential tear expression profiles of a variety of pro-inflammatory cytokines in the POAG patients as compared to the CG. Furthermore, a statistically significant increased IL-6 concentration was significantly detected in the aqueous humor and plasma samples of POAG patients respect to the CG, reinforcing the role of INF in glaucoma neurodegeneration. 

The RGCs undergo apoptosis (AP) in the POAG course [69]. In fact, the AP of the RGCs has been pointed out as an early marker of glaucoma neurodegeneration [70]. In the present work, we identified apoptotic molecules in biological samples from POAG patients and healthy CG individuals. First, we looked for CASP3, which is a cysteine-aspartyl protease encoded by the *CASP3* gene that crucially mediates the activation cascade of caspases that, in turns, is responsible for the programmed cell death. At the onset of AP, CASP 3 proteolytically cleaves poly (ADP-ribose) polymerase (PARP) and initiates the sequence of events leading to cell death. Under normal conditions, major PARP-1 function is DNA repair in response to a variety of cellular stresses. Furthermore, in response to DNA damage, PARP-1 activation is a pivotal mechanism to keep cell homeostasis or to trigger AP. Cleavage of PARP-1 by the above caspases results in the formation of two fragments: 85-kD catalytic fragment and 24-kD DNA-binding domain. Regarding these two important apoptotic players, when comparing the POAG group with their counterparts, elevated CASP3 and PARP-1 expression was detected in aqueous humor by Western blot and immunoblotting assays. These findings were also analyzed in relation to glaucoma risk factors and clinical parameters, which are essential to enhance our understanding of the pathogenic mechanisms of POAG. The endpoint of POAG neuroprotection is to prevent apoptotic RGCs death and irreversible loss of the ONFs. Trying to inhibit apoptosis, two approaches have been proposed: (1) to promote the cell survival pathways by using the brimonidine, and (2) to block the apoptotic cascade by using caspase inhibitors, such as the calpeptin [71].

Neurodegeneration is a hallmark in POAG. Some relevant pathways induce upregulation of proapoptotic gene expression, and in turn, downregulation of neuroprotective factors [68]. Neurotrophins are involved in the survival of neurons and neuroglial cells. BDNF is a neurotrophin involved in the synaptic function/plasticity of adult neurons by binding to the high-affinity receptor tyrosine kinase B (TrkB). BDNF showed significantly lower values in our cohort of POAG patients than in the CG. As BDNF may trigger a wide variety of neutroprotective mechanisms, we may hypothesize that BDNF may benefit the glaucoma course by acting as anti-apoptotic, as well as anti-autophagic molecule, to strengthen cell survival. In this context, the fatty acid amide hydrolase inhibitors and endocannabinoid system were investigated as glaucoma neuroprotectans [72]. An interesting review [73] focused on endogenous neuroprotective factors involved in RGCs survival, covering new therapeutic strategies, as the ones suggested herein. In a similar manner, recent reports discussed promising neuroprotectant candidates for preventing glaucomatous RGC death [74,75]. It is obvious that pharmacological glaucoma neuroprotection represents a stimulating field of knowledge in the pursuit for brand-new therapies for POAG.

Pivotal neurotransmitters to a wide variety of CNS functions are the serotonin and dopamine. In our cohort, both neurotransmitters were significantly reduced in plasma samples of the POAG patients as compared to the CG. In a chronic glaucoma model in the rat, Zhou et al. [76] demonstrated the protective effects of the serotonin receptor agonists on the RGCs through the regulation of the release of γ-aminobutyric acid (GABA). Dopamine is a neurotransmitter of the catecholamine family that exerts its functions by binding to 5 different types of receptors. Epidemiological and experimental studies pointed to dopamine receptor agonists as having relevant roles on IOP homeostasis, with unquestionable implications for POAG management [77]. However, the precise role of excitatory amino acids in glaucoma is not yet fully investigated.

Overall, a major challenge in the clinical practice is to address a personalized medicine [78]. In ophthalmology, this issue is underpinned by a wider scientific knowledge of the cellular and molecular basis of eye diseases. We deal with improving current information about the clinical and molecular genetics of POAG. In the midst of it all, theranostics is a newly proposed terminology evolving outstanding processes of customized diagnostic/therapy for patients, of unquestionably value in health [79,80]. Therefore, personalized medicine in glaucoma patients may be strengthened by theranostics technology that may enable the individual diagnosis and treatment strategies. In our case, the clinical, molecular-genetic candidate biomarkers described herein may potentially lead to the development of efficient imaging technologies and glaucoma therapies for better eye care.

Our study has a series of strengths. POAG diagnoses were done by ophthalmologists of the glaucoma sections, which reinforces validity. Next, is the collecting data directly from the medical history. Finally, glaucoma theranostics as a useful procedure for better eye and vision care is introduced for the first time.

In contrast, some study limitations have also been assumed. Data aimed to document personal, familial, medical, and molecular characteristics rather than disease progression. False-negative diagnoses (resulting in underestimation of POAG cases) or false-positive diagnoses, escape our control. False-positive POAG cases were noticeably reduced by the validation strategies. Classical risk factors, such as smoking and drinking habits, and physical activity were not available. Some missing data were unrecoverable from the clinical history. Statistical tests were performed for the two eyes of each participant, taking into consideration that only the initial glaucoma stage is enclosed, and that the correlation between the RE and the LE data is expected to be high. However, it has to be taken into account that an overstatement of the statistical power can appear in certain data, and this should be taken into account when interpreting the results.

Presumably, supra-molecular targeted theranostics, as suggested in the present work, may play pivotal roles for better managing POAG patients. 

## 5. Conclusions

Glaucoma is a challenging sigh threatening disease for which an incomplete knowledge remains of the cellular and molecular pathologic mechanisms involving initiation and progression stages. Glaucomatous visual disability and blindness are still irreversible. Advancing diagnostic and therapeutic options, as proposed herein via the theranostic possibilities, will enable ophthalmologists to personalize the earliest diagnosis and therapy based on outstanding specific molecules that each patient may have, to better eye and vision care in POAG patients.

Chronic IOP elevation, leading to optic nerve degeneration (coinciding with specific risk factors), provoked in its course, important changes in a variety of reactive molecules that have been found in diverse body fluids (tears, aqueous humor, plasma) during the present study. 

We have successfully identified the following: MDA, IL6, IL-8, CASP3, PARP-1, and BDNF. All these can be considered potential molecular biomarkers for POAG diagnosis, as well as for the future design of new and more efficient POAG diagnostic tools and biotherapies.

## Figures and Tables

**Table 1:** Inclusion and exclusion characteristics for the study subjects.

Inclusion	Exclusion
Individuals aged 40 years or more	Individuals under 40 years of age
Accurate POAG diagnosis (initial stage) for the corresponding group of participants.	Other glaucoma type than the POAG or glaucoma suspects.
Healthy individuals for the CG of participants	Patients suffering other eye diseases than POAG, or systemic disorders.
	Patients under treatment that may interfere with the results of the study.
	Laser and/or eye surgery in the previous 12 months.
Complete and precise data at the medical history.	Incomplete and/or confounding data. Other diagnosis or procedures that do not fit with the study purpose. Impossibility to obtain a complete and thorough clinical history

POAG: primary open-angle glaucoma; CG: control group.

## Abbreviations

5HT:	5-hydroxytriptamine
AA:	alpha-agonists
ADP	adenosine diphosphate
AP:	apoptosis
BB:	beta-blockers
BCVA:	best corrected visual acuity
BDNF:	brain-derived neurotrophic factor
BMB:	biochemical and molecular biomarkers
BMC:	biomicroscopy
CAI:	carbonic anhydrase inhibitors
CAS3:	caspase-3
CCT:	central corneal thickness
CG:	control group
DARC:	detection of apoptosing retail cells
ELISA:	enzyme-linked immunosorbent assays
FLIO:	fluorescence lifetime imaging ophthalmoscope
GABA:	y-aminobutyric acid
GM-CSF:	colony-stimulating factor
GPx:	glutathione peroxidase
GTP:	guanosine triphosphatase
H_2_O_2_:	hydrogen peroxide
HPLC:	high performance liquid chromatography
IFγ:	interferon gamma
IL:	interleukin
IOP:	intraocular pressure
LE:	left eye
MD:	mean derivation
MDA:	malondialdehyde
MD:	mediterranean diet
MIGS:	minimally invasive glaucoma surgery
NF-κB:	nuclear factor kappa B
NO:	nitric oxide
NPDS:	non-perforating deep sclerectomy
OCT:	optical coherence tomography
OCTA:	optical coherence tomography angiography
OF:	ocular fundus
OH:	hydroxyl radical
ORAC:	oxygen-radical absorbance capacity method
OS:	oxidative stress
PA:	prostaglandin analogues
PARP1:	poly ADP-ribose polymerase-1
POAG:	primary open-angle glaucoma
RE:	right eye
RGC:	retinal ganglion cells
RNFL:	retinal nerve fiber layer
ROS:	reactive oxygen species
SD:	standard derivation
SOD:	superoxide dismutase
TAC:	total antioxidant capacity
TBARS:	thiobarbituric acid reactive substances
TM:	trabecular meshwork
TNFα:	tumour necrosis factor alpha
TrkB:	tyrosine kinase B
VEGF:	vascular endothelial growth factor
VF:	visual field

## References

[1] Flaxman S.R., Bourne R.R.A., Resnikoff S., Ackland P., Braithwaite T., Cicinelli M.V., Das A., Jonas J.B., Keeffe J., Kempen J.H. (2017). On behalf of the Vision Loss Expert Group of the Global Burden of Disease Study† et al., Vision loss expert group of the global burden of disease study. Global causes of blindness and distance vision impairment 1990–2020: A systematic review and meta-analysis. Lancet Glob. Health.

[2] Bourne R.R.A. (2020). Vision 2020: Where are we?. Curr. Opin. Ophthalmol..

[3] Pinazo-Durán M.D., Muñoz-Negrete F.J., Sanz-González S.M., Benítez-del-Castillo J., Giménez-Gómez R., Serrano M., Zanón-Moreno V., García-Medina J.J. (2020). The role of neuroinflammation in the pathogenesis of glaucoma neurodegeneration. Prog. Brain Res..

[4] Lee R.M.H., Bouremel Y., Eames I., Brocchini S., Khaw P.T. (2020). Translating Minimally Invasive Glaucoma Surgery Devices. Clin. Transl. Sci..

[5] Occhiutto M.L., Maranhão R.C., Costa V.P., Konstas A.G. (2020). Nanotechnology for Medical and Surgical Glaucoma Therapy-A Review. Adv. Ther..

[6] Sheybani A., Scott R., Samuelson T.W., Kahook M.Y., Bettis D.I., Ahmed I.I.K., Stehpehs J.D., Kent D., Ferguson T.J., Herndon L.W. (2020). Open-Angle Glaucoma: Burden of Illness, Current Therapies, and the Management of Nocturnal IOP Variation. Ophthalmol. Ther..

[7] Medeiros F.A. (2017). Biomarkers and Surrogate Endpoints: Lessons Learned From Glaucoma. Invest. Ophthalmol. Vis. Sci..

[8] Yohannan J., Boland M.V. (2017). The Evolving Role of the Relationship between Optic Nerve Structure and Function in Glaucoma. Ophthalmology.

[9] Izzotti A., Bagnis A., Saccá S. (2006). The role of oxidative stress in glaucoma. Mutat. Res..

[10] Erdurmuş M., Yağcı R., Atış Ö., Karadağ R., Akbaş A., Hepşen I.F. (2011). Antioxidant status and oxidative stress in primary open angle glaucoma and pseudoexfoliative glaucoma. Curr. Eye Res..

[11] Pinazo-Durán M.D., Gallego-Pinazo R., García-Medina J.J., Zanón-Moreno V., Nucci C., Dolz-Marco R., Martínez-Castillo S., Galbis-Estrada C., Marco-Ramírez C., López-Gálvez M.I. (2014). Oxidative stress and its downstream signaling in aging eyes. Clin. Interv. Aging..

[12] Saccà S.C., Gandolfi S., Bagnis A., Manni G., Damonte G., Traverso C.E., Izzotti A. (2016). From DNA damage to functional changes of the trabecular meshwork in aging and glaucoma. Ageing Res. Rev..

[13] Sathiyanathan P., Tay C.Y., Stanton L.W. (2017). Transcriptome analysis for the identification of cellular markers related to trabecular meshwork differentiation. BMC Genom..

[14] Benitez-Del-Castillo Sánchez J., Morillo-Rojas M.D., Galbis-Estrada C., Pinazo-Duran M.D. (2017). Determination of inmune response and inflammation mediators in tears: Changes in dry eye and glaucoma as compared to healthy controls. Arch. Soc. Esp. Oftalmol..

[15] Mayordomo-Febrer A., Lopez-Murcia M., Morales-Tatay J.M., Monleón-Salvadó D., Pinazo-Durán M.D. (2015). Metabolomics of the aqueous humor in the rat glaucoma model induced by a series of intracamerular sodium hyaluronate injection. Exp. Eye Res..

[16] Lanza M., Benincasa G., Costa D., Napoli C. (2019). Clinical role of epigenetics and network analysis in eye diseases. A translational science review. J. Ophthalmol..

[17] Rossi C., Cicalini I., Cufaro M.C., Agnifili L., Mastropasqua L., Marchisio M., de Laurenzi V., del Boccio P., Pieragostino D. (2019). Multiomics approach for studying tears in treatment naïve glaucoma patients. Int. J. Mol. Sci..

[18] Izzotti A., Cecaroli C., Longobardi G.M., Micale T.R., Pulliero A., La Maestra S., Saccá S. (2015). Molecular damage in glaucoma: From anterior to posterior eye segment. The microRNA role. Microma.

[19] Altman R.B. (2012). Translational bioinformatics: Linking the molecular world to the clinical world. Clin. Pharmacol. Ther..

[20] Kievit F.M., Zhang M. (2011). Cancer nanotheranostics: Improving imaging and therapy by targeted delivery across biological barriers. Adv. Mater..

[21] Zinnhardt B., Belloy M., Fricke I.B., Orije J., Guglielmetti C., Hermann S., Wagner S., Schäfers M., van der Linden A., Jacobs A.H. (2019). Molecular Imaging of Immune Cell Dynamics During De- and Remyelination in the Cuprizone Model of Multiple Sclerosis by [18F]DPA-714 PET and MRI. Theranostics.

[22] Vaz S.C., Oliveira F., Herrmann K., Veit-Haibach P. (2020). Nuclear medicine and molecular imaging advances in the 21st century. Br. J. Radiol..

[23] Liu Z. (2020). Featuring advanced translational strategies: Principles, techniques, devices and applications. Cancer Lett..

[24] Doupe M.B., Poss J., Norton P.G., Garland A., Dik N., Zinnick S., Lix L.M. (2018). How well does the minimum data set measure healthcare use? A validation study. BMC Health Serv. Res..

[25] Mills R.P., Budenz D.L., Lee P.P., Noecker R.J., Walt J.G., Siegartel L.R., Evans S.J., Doyle J.J. (2006). Categorizing the stage of glaucoma from pre-diagnosis to end-stage disease. Am. J. Ophthalmol..

[26] Hodapp E., Parrish R.K. (1993). II Anderson D.R. Clinical Decisions in Glaucoma.

[27] Estruch R., Martínez-González M.A., Corella D., Salas-Salvadó J., Fitó M., Chiva-Blanch G., Fiol M., Gómez-Gracia E., Arós F., Lapetra L. (2006). PREDIMED Study Investigators Effects of a Mediterranean-style diet on cardiovascular risk factors: A randomized trial. Ann. Intern. Med..

[28] Lima-Oliveira G., Volanski W., Lippi G., Picheth G., Guidi G.C. (2017). Pre-analytical phase management: A review of the procedures from patient preparation to laboratory analysis. Scand. J. Clin. Lab. Investig..

[29] Zanon-Moreno V., Marco-Ventura P., Lleo-Perez A., Pons-Vazquez S., Garcia-Medina J.J., Vinuesa-Silva I., Moreno-Nadal M.A., Pinazo-Duran M.D. (2008). Oxidative stress in primary open-angle glaucoma. J. Glaucoma.

[30] Pinazo-Durán M.D., Galbis-Estrada C., Pons-Vázquez S., Cantú-Dibildox J., Marco-Ramírez C., Benítez-del-Castillo J. (2013). Effects of a nutraceutical formulation based on the combination of antioxidants and ω-3 essential fatty acids in the expression of inflammation and immune response mediators in tears from patients with dry eye disorders. Clin. Interv. Aging.

[31] Uchiyama M., Mihara M. (1978). Determination of malonaldehyde precursor in tissues by thiobarbituric acid test. Anal. Biochem..

[32] Pinazo-Duran M.D., Shoaie-Nia K., Zanon-Moreno V., Sanz-Gonzalez S.M., del Castillo J.B., Garcia-Medina J.J. (2018). Strategies to Reduce Oxidative Stress in Glaucoma Patients. Curr. Neuropharmacol..

[33] Csallany A.S., der Guan M., Manwaring J.D., Addis P.B. (1984). Free Malonaldehyde Determination in Tissues by High-Performance Liquid Chromatography. Anal. Biochem..

[34] Nucci C., Di Pierro D., Varesi C., Ciuffoletti E., Russo R., Gentile R., Cedrone C., Pinazo-Duran M.D., Coletta M., Mancino R. (2013). Increased malondialdehyde concentration and reduced total antioxidant capacity in aqueous humor and blood samples from patients with glaucoma. Mol. Vis..

[35] Zanon-Moreno V., Garcia-Medina J.J., Gallego-Pinazo R., Vinuesa-Silva I., Moreno-Nadal M.A., Pinazo-Duran M.D. (2009). Antioxidant status modifications by topical administration of dorzolamide in primary open-angle glaucoma. Eur. J. Ophthalmol..

[36] Zanon-Moreno V., Asensio-Marquez E.M., Ciancotti-Oliver L., García Medina J.J., Sanz P., Ortega-Azorin C., Pinazo-Duran M.D., Ordovás M., Corella D. (2013). Effects of polymorphisms in vitamin E-, vitamin C-, and glutathione peroxidase-related genes on serum biomarkers and associations with glaucoma. Mol. Vis..

[37] Li X., Franke A.A. (2009). Fast HPLC–ECD analysis of ascorbic acid, dehydroascorbic acid and uric acid. J. Chromatogr. B Analyt. Technol. Biomed. Life Sci..

[38] Zanon-Moreno V., Ciancotti-Olivares L., Asencio J., Sanz P., Ortega-Azorin C., Pinazo-Duran M.D., Corella D. (2011). Association between a SLC23A2 gene variation, plasma vitamin C levels, and risk of glaucoma in a Mediterranean population. Mol. Vis..

[39] Zanón-Moreno V., Pons S., Gallego-Pinazo R., García-Medina J.J., Vinuesa I., Vila-Bou V., Pinazo-Durán M.D. (2008). Involvement of nitric oxide and other molecules with redox potential in primary open angle glaucoma. Arch. Soc. Esp. Oftalmol..

[40] Benitez-Del-Castillo J., Cantu-Dibildox J., Sanz-González S.M., Zanón-Moreno V., Pinazo-Duran M.D. (2019). Cytokine expression in tears of patients with glaucoma or dry eye disease: A prospective, observational cohort study. Eur. J. Ophthalmol..

[41] Zanon-Moreno V., Garcia-Medina J.J., Zanon-Viguer V., Moreno-Nadal M.A., Pinazo-Duran M.D. (2009). Smoking, an additional risk factor in elder women with primary open-angle glaucoma. Mol. Vis..

[42] Chandra D., Tang D.G. (2009). Detection of apoptosis in cell-free systems. Methods Mol. Biol. Clifton N. J..

[43] Lowry O.H., Rosebrough N.J., Farr A.L., Randall R.J. (1951). Protein Measurement with the Folin Phenol Reagent. J. Biol. Chem..

[44] Naegelin Y., Dingsdale H., Säuberli K., Schädelin S., Kappos L., Barde Y.A. (2018). Measuring and Validating the Levels of Brain-Derived Neurotrophic Factor in Human Serum. eNeuro.

[45] Ali S.F., Newport G.D., Scallet A.C., Binienda Z., Ferguson S.A., Bailey J.R., Paule M.G., Slikker W. (1993). Oral administration of 3,4-methylenedioxymethamphetamine (MDMA) produces selective serotonergic depletion in the nonhuman primate. Neurotoxicol. Teratol..

[46] Gordon M.O., Beiser J.A., Brandt J.D., Heuer D.K., Higginbotham E.J., Johnson C.A., Keltner J.L., Miller J.P., Parrish R.K., Wilson M.R. (2002). The Ocular Hypertension Treatment Study: Baseline factors that predict the onset of primary open-angle glaucoma. Arch. Ophthalmol..

[47] Hollands H., Johnson D., Hollands S., Simel D.L., Jinapriya D., Sharma S. (2013). Do findings on routine examination identify patients at risk for primary open-angle glaucoma? The rational clinical examination systematic review. JAMA.

[48] Kreft D., Doblhammer G., Guthoff R.F., Frech S. (2019). Prevalence, incidence, and risk factors of primary open-angle glaucoma—A cohort study based on longitudinal data from a German public health insurance. BMC Public Health.

[49] Vianna J.R., Danthurebandara V.M., Sharpe G.P., Hutchison D.M., Belliveau A.C., Shuba L.M., Nicolela M.T., Chauhan B.C. (2015). Importance of Normal Aging in Estimating the Rate of Glaucomatous Neuroretinal Rim and Retinal Nerve Fiber Layer Loss. Ophthalmology.

[50] Liu S.A., Zhao Z.N., Sun N.N., Han Y., Chen J., Fan Z.G. (2018). Transitions of the Understanding and Definition of Primary Glaucoma. Chin. Med. J. Engl..

[51] Kang J.H., Pasquale L.R., Willett W., Rosner B., Egan K.M., Faberowski N., Hankinson S.E. (2003). Antioxidant intake and primary open-angle glaucoma: A prospective study. Am. J. Epidemiol..

[52] Bussel I.I., Aref A.A. (2014). Dietary factors and the risk of glaucoma: A review. Ther. Adv. Chronic Dis..

[53] Abreu-Reyes J.A., Álvarez-Luis D., Arteaga-Hernández V., Sánchez-Mendez M., Abreu-González R. (2017). Mediterranean diet adherence of patients with primary open-angle glaucoma. Arch. Soc. Esp. Oftalmol..

[54] Sofi F., Cesari F., Abbate R., Gensini G.F., Casini A. (2008). Adherence to Mediterranean diet and health status: Meta-analysis. BMJ.

[55] Tuulonen A., Forsman E., Hagman J., Harju M., Kari O., Lumme P., Luodonpää M., Määttä M., Saarela V., Vaajanen A. (2015). [Update on Current Care Guideline: Glaucoma]. Duodecim.

[56] Nucci C., Martucci A., Giannini C., Morrone L.A., Bagetta G., Mancino R. (2018). Neuroprotective agents in the management of glaucoma. Eye.

[57] Konstantakopoulou E., Gazzard G., Vickerstaff V., Jiang Y., Nathwani N., Hunter R., Ambler G., Bunce C., LiGHT Trial Study Group (2018). The Laser in Glaucoma and Ocular Hypertension (LiGHT) trial. A multicentre randomised controlled trial: Baseline patient characteristics. Br. J. Ophthalmol..

[58] Pinazo-Durán M.D., Zanón-Moreno V., García-Medina J.J., Gallego-Pinazo R. (2013). Evaluation of presumptive biomarkers of oxidative stress, immune response and apoptosis in primary open-angle glaucoma. Curr. Opin. Pharmacol..

[59] Kambayashi Y., Binh N.T., Asakura H.W. (2009). Efficient assay for total antioxidant capacity in human plasma using a 96-well microplate. J. Clin. Biochem. Nutr..

[60] Lymperaki E., Tsikopoulos A., Makedou K., Paliogianni E., Kiriazi L.M., Charisi C., Vagdatli E. (2015). Impact of iron and folic acid supplementation on oxidative stress during pregnancy. J. Obstet. Gynaecol..

[61] Guo C., Sun L., Chen X., Zhang D. (2013). Oxidative stress, mitochondrial damage and neurodegenerative diseases. Neural Reg. Res..

[62] Bhatti J.S., Bhatti G.K., Reddy P.H. (2017). Mitochondrial dysfunction and oxidative stress in metabolic disorders—A step towards mitochondria based therapeutic strategies. Biochim. Biophys. Acta Mol. Basis Dis..

[63] Pinazo-Durán M.D., Zanón-Moreno V., Gallego-Pinazo R., García-Medina J.J. (2015). Oxidative stress and mitochondrial failure in the pathogenesis of glaucoma neurodegeneration. Prog. Brain Res..

[64] Saccà S.C., Pascotto A., Camicione P., Capris P., Izzotti A. (2005). Oxidative DNA damage in the human trabecular meshwork. Clinical correlation in patients with primary open-angle glaucoma. Arch. Ophthalmol..

[65] Murakami Y., Ishikawa K., Nakao S., Sonoda K.H. (2020). Innate immune response in retinal homeostasis and inflammatory disorders. Prog. Retin. Eye Res..

[66] Tezel G., Yang X., Luo C., Peng Y., Sun S.L., Sun D. (2007). Mechanisms of immune system activation in glaucoma: Oxidative stress-stimulated antigen presentation by the retina and optic nerve head glia. Investig. Ophthalmol. Vis. Sci..

[67] Yerramothu P., Vijay A.K., Willcox M.D.P. (2018). Inflammasomes, the eye and anti-inflammasome therapy. Eye.

[68] Zanon-Moreno V., Raga-Cervera J., García-Medina J.J., Benitez-del-Castillo J., Vinuesa-Silva I., Torregrosa S., Pinazo-Durán M.D. (2018). New horizons for glaucoma therapy. I: Neuroinflammation and inflammasomes. Arch. Soc. Esp. Oftalmol..

[69] Cordeiro M.F., Guo L., Luong V., Harding G., Wang W., Jones H.E., Moss S.E., Sillito A.M., Fitzke F.W. (2004). Real-time imaging of single nerve cell apoptosis in retinal neurodegeneration. Proc. Natl. Acad. Sci. USA.

[70] Gauthier A.C., Liu J. (2017). Epigenetics and Signaling Pathways in Glaucoma. Biomed. Res. Int..

[71] Vasudevan S.K., Gupta V., Crowston J.G. (2011). Neuroprotection in glaucoma. Indian J. Ophthalmol..

[72] Pietrucha-Dutczak M., Amadio M., Govoni S., Lewin-Kowalik J., Smedowski A. (2018). The Role of Endogenous Neuroprotective Mechanisms in the Prevention of Retinal Ganglion Cells Degeneration. Front. Neurosci..

[73] Garcia-Medina J.J., Rubio-Velazquez E., Foulquie-Moreno E., Casaroli-Marano R.P., Pinazo-Durán M.D., Zanón-Moreno V., del-Rio-Vellosillo M. (2020). Update on the effects of antioxidants on diabetic. Retinopathy: In Vitro Experiments, Animal Studies and Clinical Trials. Antioxidants.

[74] Fernández-Albarral J.A., de Hoz R., Ramirez A.I., López-Cuenca I., Salobrar-García E., Pinazo-Durá M.D., Ramírez J.M., Salazar J.J. (2020). Beneficial effects of saffron (*Crocus sativus* L.) in ocular pathologies, particularly neurodegenerative retinal diseases. Neural Regen. Res..

[75] Fernández-Albarral J.A., Ramírez A.I., de Hoz R., López-Villarín N., Salobrar-García E., López-Cue I., Licastro E., Inarejos-García A.M., Almodóvar P., Pinazo-Durán M.D. (2019). Neuroprotective and Anti-Inflammatory Effects of a Hydrophilic Saffron Extract in a Model of.Glaucoma. Int. J. Mol. Sci..

[76] Zhou X., Li G., Zhang S., Wu J. (2019). 5-HT1A Receptor Agonist Promotes Retinal Ganglion Cell Function by Inhibiting OFF-Type Presynaptic Glutamatergic Activity in a Chronic Glaucoma Model. Front. Cell. Neurosci..

[77] Pesscosolido N., Parisi F., Russo P., Buomprisco G., Nebbioso M. (2013). Role of dopaminergic receptors in glaucomatous disease modulation. BioMed Res. Int..

[78] Porter L.F., Black G.C. (2014). Personalized ophthalmology. Clin. Genet..

[79] Kubelick K.P., Snider E.J., Ethier R., Emelianov S. (2019). Development of a stem cell tracking platform for ophthalmic applications using ultrasound and photoacoustic imaging. Theranostics.

[80] Shiri I., Abdollahi H., Atashzar M.R., Rahmim A., Zaidi H. (2020). A theranostic approach based on.radiolabeled antiviral drugs, antibodies and CRISPR-associated proteins for early detection and treatment of SARS-CoV-2 disease. Nucl. Med. Commun..

